# Study on the efficiency of virtual reality in the treatment of alcohol use disorder: study protocol for a randomized controlled trial: *E-Reva*

**DOI:** 10.1186/s13063-024-08271-x

**Published:** 2024-06-27

**Authors:** Fanny Nègre, Maud Lemercier-Dugarin, Romain Gomet, Antoine Pelissolo, Eric Malbos, Lucia Romo, El-Hadi Zerdazi

**Affiliations:** 1 Laboratoire CLIPSYD, Université Paris Nanterre, 92000 Nanterre, France; 2 DMU IMPACT, APHP, Hôpitaux Universitaires Henri-Mondor, Hôpital Albert-Chenevier, Service d’addictologie, 94010 Créteil, France; 3 Université de Caen Normandie, LPCN UR, 7452, F-14000 Caen, France; 4 DMU IMPACT, AP-HP, Université Paris-Est-Créteil (UPEC), Hôpitaux Universitaires Henri-MondorService de Psychiatrie, 94000 Créteil, France; 5 Université Paris-Est Créteil, INSERM U995, IMRB, Translational Neuropsychiatry Laboratory, 94010 Créteil, France; 6 Psychiatry Service of Pr Lançon, CHU de Sainte Marguerite, Marseille, France; 7 Institut Fresnel Aix-Marseille Université, CNRS, Ecole Centrale Marseille, UMR, 72490 Marseille, France; 8 APHP, Hôpital Universitaire Raymond Poincaré, 92380 Garches, France; 9 Université Paris Saclay, INSERM CESP, 1018 UPS, 94807 Villejuif, France

**Keywords:** Virtual reality, VR, Alcohol use disorder, AUD, Protocol, Treatment, CBT, RCT

## Abstract

**Context:**

According to the World Health Organization, alcohol is a major global public health problem, leading to a significant increase in illness and death. To treat alcohol use disorders, new therapeutic tools are being promoted, among which virtual reality (VR) shows promise. Previous research has demonstrated the efficacy of VR in reducing alcohol cravings in patients, but there is a lack of data on its effectiveness in maintaining abstinence or reducing consumption in recently abstinent individuals.

The E-Reva study aims to compare the efficacy of a treatment strategy combining virtual reality cue exposure therapy (VR-CET) and cognitive behavioral therapy (CBT) with conventional CBT in reducing alcohol consumption and craving in patients with alcohol use disorder (AUD). In addition to this primary objective, the study will compare the effects of VR-CET combined with CBT on anxiety, depression, rumination, and feelings of self-efficacy versus conventional CBT.

**Methods:**

This prospective randomized controlled trial will be conducted over 8 months in four addiction departments in France. It includes two parallel groups: i) the VR-CET + CBT group, and ii) the CBT-only group, which serves as a control group.

Participants will be recruited by the investigating doctor in the addiction centers. The sample will consist of 156 patients diagnosed with AUD and abstinent for at least 15 days.

Both treatment groups will participate in four group CBT sessions followed by four individual sessions: i) the VR-CET group will be exposed to virtual environments associated with alcohol-related stimuli, ii) the CBT-only group will receive traditional CBT sessions. After completion of the 8 sessions, patients will be followed up for 6 months. The primary outcome is the cumulative number of standard drinks consumed at 8 months, assessed using the TLFB method.

**Discussion:**

Despite the promise of VR-CET to reduce the desire to drink, the effect on alcohol consumption remains uncertain in the existing literature. Our protocol aims to address the limitations of previous research by increasing sample size, targeting consumption reduction, and incorporating neutral environments. E-Reva aims to enrich the literature on the use of VR in the treatment of AUD and open new perspectives for future interventions.

**Trial registration:**

ClinicalTrials.gov ID NCT06104176, Registered 2023/11/13 (https://clinicaltrials.gov/study/NCT06104176?id=NCT06104176&rank=1).

N° IDRCB: 2022-A02797-36.

Protocol version 1.0, 12/05/2023.

**Supplementary Information:**

The online version contains supplementary material available at 10.1186/s13063-024-08271-x.

## Introduction

Responsible for nearly 3.3 million deaths per year, alcohol has been a worldwide public health problem for years [1, 2]. The ethanol (ethyl alcohol) in alcohol is responsible for numerous disorders: cognitive and behavioral disorders, certain types of cancer, cardiovascular disease, higher risk of cirrhosis and pancreatitis, and trauma from violence and road or work accidents [3]. Excessive alcohol consumption therefore induces neurobiological and behavioral changes, creating a breeding ground for the development of alcohol use disorder (AUD). According to the 5th version of the Diagnostic and Statistical Manual of Mental Disorders, AUD is defined as "a set of behavioral, cognitive, and physiological phenomena that result in progressive disinvestment from other activities" [4]. Despite the harmful physical and emotional effects of excessive alcohol use, many people find it difficult to reduce or maintain abstinence over the long term [5].

Craving is defined as "a compelling need to consume the substance" that can lead to compulsive substance seeking and consumption-related behaviors. Craving elicits psychophysiological, emotional, cognitive, and behavioral responses that follow a subjective perception of the urge to drink alcohol [6]. Craving is a key element in the development and maintenance of excessive drinking and AUD [7, 8].

Treatment for AUD is based on a multidimensional approach that includes pharmaceutical, behavioral, and psychosocial interventions [7]. These treatments aim to address loss of control over use, reduce negative emotions and hyperreactivity to stress, and/or reduce craving [9]. Among these interventions, several meta-analyses conclude that CBTs are effective in treating addictive disorders, have few contraindications, and are easy to implement [10, 11].

One of the tools used in CBTs is cue exposure therapy (CET), which aims to teach craving self-management techniques through in vivo, in imagination, or multimedia exposure to substance-related stimuli [7, 12]. CET is based on the principles of deconditioning a behavior considered maladaptive to replace it with one considered more adaptive, and systematically desensitizing the subject by immersing him or her in the anxiety-provoking environment. Despite its success in treating anxiety disorders, CET has shown limited results in reducing alcohol consumption [13]. This limited effectiveness appears to be related to the ecological validity of the cues used [12, 13]. The in vivo exposures proposed in CET are costly and complex to replicate, as they require specific environments and situations (e.g., bars, nightclubs…). Consequently, exposures are often performed in the laboratory, but these experimental approaches involve artificial behavioral responses to simple stimuli, with constraints on the modification of parameters, context, and available space, which limits the complexity of behavioral interactions [8, 14].

Virtual reality exposure therapy (VR-CET) is a new therapeutic exposure technique that uses virtual reality (VR) to allow users to navigate and interact with a computer-generated (and computer-maintained) three-dimensional environment in real time through a headset [15]. The advantages of this technique are the therapist's control over the exposure, the freedom to stop exposure at any time if necessary, the possibility of adapting the virtual environment to the subject's problems, and better observation of the patient's reactions. All these elements promote greater immersion and interaction of the patient with the virtual environment than, for example, exposure in imagination, another typical technique used in CBT [16]. Moreover, technological advances in recent years have made it widely available, easily accessible, simpler to use, better tolerated, and increasingly less expensive. Several studies report promising results for VR-CET in the treatment of various mental disorders [16], including addictive disorders [17]. The use of virtual reality was motivated by the fact that many patients were reluctant to experience violent anxiety-provoking situations in vivo during CBT sessions [18]. Thus, the application of VR-CET has been extended to many other specific phobic disorders (acrophobia, arachnophobia, social phobia, claustrophobia, agoraphobia,etc.) and has shown a therapeutic effect superior to no intervention and equivalent to exposure through standard techniques [18–20]. For people suffering from excessive alcohol consumption, VR-CET has been shown to be effective in inducing and reducing craving [21]. Despite this promising advance, there remains a gap in the data on the efficacy of VR-CET in maintaining abstinence or reducing alcohol consumption, which remain the primary goals of therapeutic interventions. This raises the need for further studies to shed light on this area of research. The primary objective of this study is to evaluate the efficacy of a treatment strategy combining VR-CET and CBT on reducing the cumulative number of standard drinks (sd) of alcohol at 8 months after enrollment. Secondary objectives are to evaluate the efficacy of the same treatment strategy on reducing alcohol craving, anxiety, depression, and rumination, and on increasing self-efficacy at 8 months. This article describes, in accordance with SPIRIT standards [22] and following the CONSORT [23] declaration checklist A prospective, comparative, randomized, open-label, 8-month clinical trial (2 months of therapy followed by 6 months of follow-up) with two parallel groups: i) VR-CET + CBT, ii) CBT alone without exposure (control group). This was a national multicenter study involving four addictology centers. A total of 156 patients diagnosed with AUD according to DSM-5 criteria were included.

## Methods/Design

### Study setting and population

Study data will be collected in day hospitals (DH) within the addiction departments of public hospitals. For this research, four centers in the Paris area will be involved in recruiting patients. The four recruiting centers are: 1) Department of Addictions—Hôpital Albert Chenevier—CHU Henri Mondor—APHP – Créteil, 2) Department of Addictions—Hôpital Fernand Widal—Nord—Université de Paris—APHP – Paris, 3) Department of Addictions—Centre hospitalier des Quatre Villes – Sèvres, 4) Department of Addictions—Tolbiac Day Clinic – Paris. Recruitment will be done by the investigating physician at the time of admission to the DH during an individual consultation. If the patient is eligible for the study, the full protocol will be presented and reviewed during an individual consultation at the DH enrollment visit.

The following inclusion criteria apply: i) 18 to 80 years of age at the time of enrolment; ii) patients with a diagnosis of AUD according to DSM-5 criteria (American Psychiatric Association, 2013); iii) patients who had been abstinent for at least 15 days; iv) patients who can speak, understand and read French; v) patients who have signed an informed consent form and who are affiliated to a health insurance plan.

Subjects are not included if they are: i) adults under legal protection or deprived of liberty by judicial or administrative decision; ii) pregnant or breast-feeding women; iii) patients with decompensated psychiatric disorders (psychotic disorders, mood disorders and anxiety disorders); iv) patients relapsing from their AUD; v) patients with severe cognitive impairment as defined by the MOCA Cognitive Assessment Test (score < 10) [24]; vi) visually impaired patients; vii) patients with contraindications to virtual reality exposure, due to epilepsy or history of photoparoxysmal EEG response, or vestibular disorders; viii) recent cardiovascular accident less than 3 months old, current nausea/vomiting, claustrophobia and moderate or severe myopia (greater than -3. 5 diopters); ix) patients wearing any of the following medical devices (due to the risk of interference with the virtual reality headset): pacemaker, implanted defibrillator or implanted hearing aid (non-implanted prostheses are not contraindicated if the patient agrees to remove them during VR-CET); x) participation in another study or being in the exclusion period after previous research involving human subjects; xi) and patients benefiting from State Medical Assistance (defined as access to healthcare for people in an irregular situation).

### Study design and procedure

**Fig. 1:**
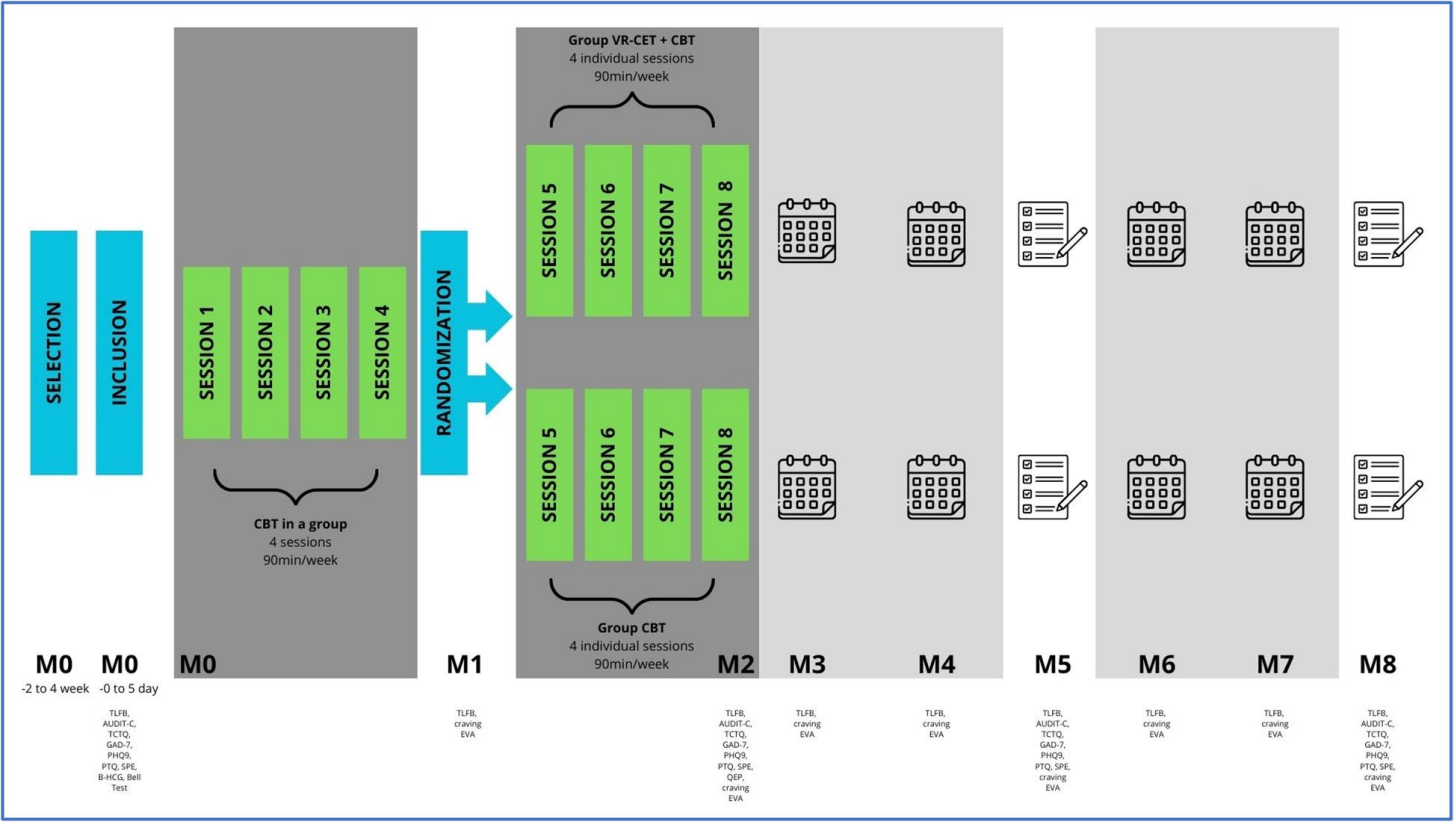
Participant timeline

This study is a multi-center, randomized, controlled trial involving two parallel groups: i) the VR-CET group, which benefits from virtual reality exposure preceded by CBT sessions, and ii) the CBT-only group (control group). Interventions take place over a 2-month (8-week) cycle, with a total of 8 sessions (Cf. Figure 1). Both groups receive 4 CBT group sessions in the first month, followed by either 4 supervised individual virtual exposure sessions for the VR-CET group, or 4 additional individual CBT sessions for the CBT-only group. Each session lasts one hour and a half. Once the 8 sessions have been completed (2 months), patients are followed up for 6 months, for a total of 8 months.

### Treatment groups

The first four CBT group sessions focus on understanding the effects of alcohol, assessing motivation to change, behavioral and emotional strategies, cognitive strategies, relapse prevention and assertiveness strategies. These sessions are conducted in a group setting by a trained psychologist and a trainee.

Participants are then randomized into two groups (M1): i) VR-CET + BT group, ii) CBT group only. Allocation is centralized, individualized and randomized according to the following factors: center, presence of pharmacological treatment to maintain alcohol abstinence (2 modalities), history of AUD treatment in the last five years (2 modalities), alcohol consumption goals (2 modalities) and severity of alcohol dependence according to DSM-5 criteria (3 modalities).

#### CBT treatment group

Individual CBT sessions (sessions 5 to 8 in the control group) are conducted by a CBT-trained investigator and focus on reviewing and adapting the strategies discussed in the group sessions. The sessions last between 30 and 90 min and are designed to develop a list of strategies adapted to the patient's daily life, addressing various environments such as the supermarket, parties, home and exposure to advertising. The patient's desire to drink is regularly assessed using a visual analog scale (VAS).

#### VR-CET treatment group

**Fig. 2:**
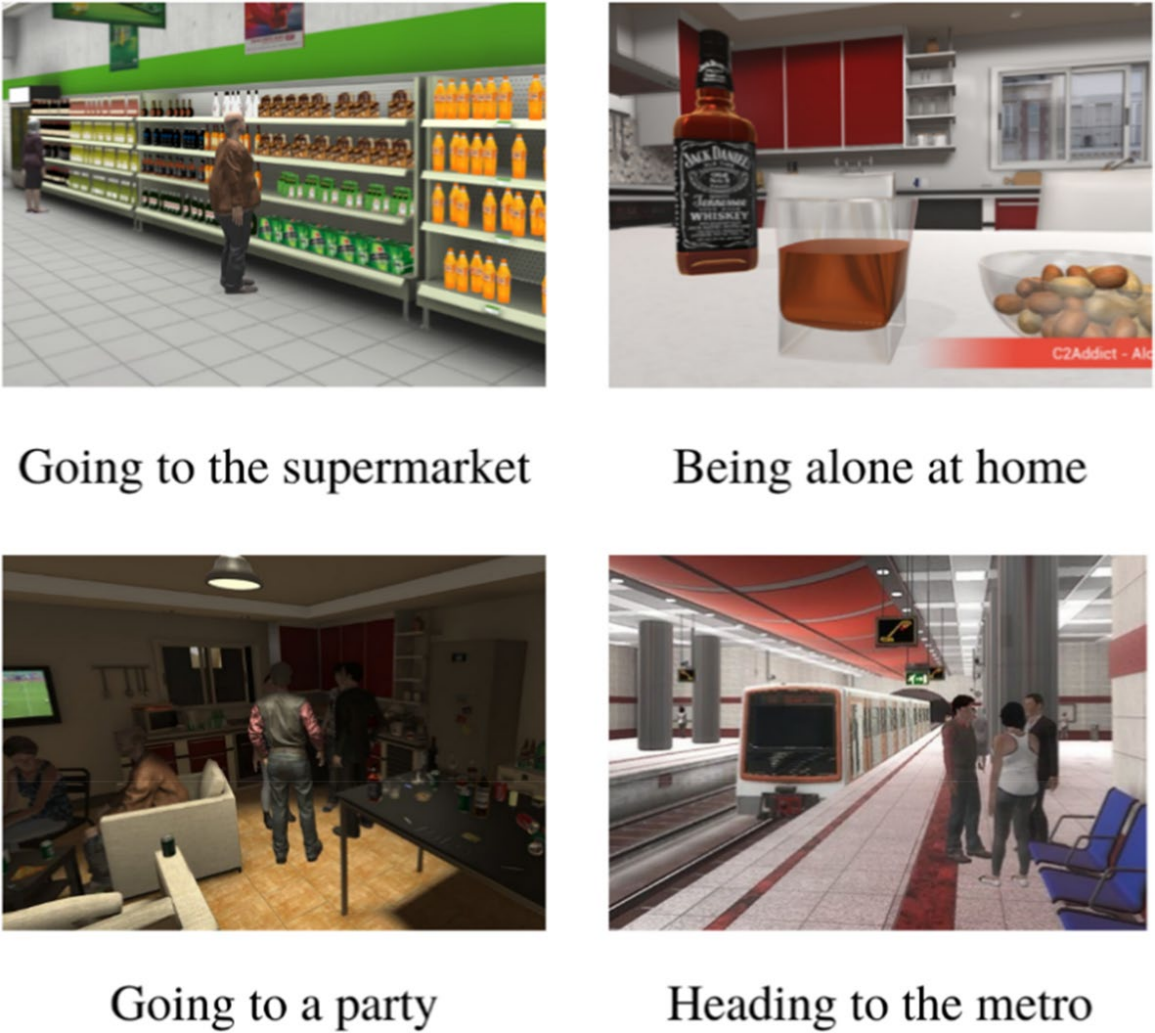
Screenshots of the virtual environments utilized in the present study

For the VR-CET group, individual exposure sessions (sessions 5 to 8) are conducted by an investigator trained in CBT. Prior to the exposure sessions, each patient will establish a hierarchy of four virtual environments: exposure to alcohol advertising, home, supermarket, and parties (cf. Figure 2) ranging from the simplest to the hardest. The duration of exposure to each virtual environment varies according to the time needed to reduce the participants’ craving. Participants move on to the next environment when a comfortable level of emotion or craving is reached, and exposure can be repeated if necessary. If craving remains high at the end of a session, it is extended. After exposure, patients benefit from a 5 to 10 min VR relaxation session. Craving is regularly assessed using a VAS before, during and at the end of each session.

### Apparatus

For the VR-CET group, the virtual reality device is as follows:

1. An HP reverb G2 virtual reality headset with 2160 × 2160 resolution per eye and position sensor integrated into the headset.
2. Two motion controllers in the form of joysticks in each hand that locate the position of the thumb, index and middle fingers in contact with the buttons. In addition to walking, the wireless joysticks enable subjects to interact with 3D objects (taking a drink, picking up a bottle, etc.). The subject thus produces equivalent movements in real time and explores synthetic situations visually.
3. Victus laptop by HP Laptot 16-s0025nf NVIDIA GeForce RTX 4060.

The software exploited to run the virtual reality environments was C2Care. In the alcohol addiction care offering, we have selected 4 environments, following a focus group in 2019, offering distinct scenarios inducing the desire to drink. The environments selected are as follows (Cf. Figure 2):

1. Heading for the metro, where there is a large alcohol advertisement;
2. Go to the supermarket and pass through the alcohol aisle;
3. Find yourself alone at home with a bottle of alcohol on the table;
4. Going to a party.

### Criteria for discontinuing or modifying allocated interventions

Participants can end their participation in the research at any time, and the investigator may do so for safety reasons or in the best interest of the participant. Temporary break in participation may occur in cases of persistent psychological distress, trauma during sessions, or discomfort related to virtual reality. Early termination may occur if inclusion criteria are not met. Pregnancy, protective measures, or enrollment in another study will result in definitive dropout. Absence from group sessions (50%) may lead to exclusion.

### Evaluation outcomes

#### Primary outcome

The primary outcome is the cumulative number of standard drinks consumed (in grams of pure alcohol) at 8 months, as reported by patients. As a reminder, in France, 1 standard drink = 10 g of pure alcohol = 1 unit of alcohol [25]. Alcohol consumption is estimated retrospectively by patients every month using the Timeline Follow-Back (TLFB) method, a widely used method that has demonstrated good psychometric properties and superiority over free recall [26]. This method allows retrospective estimation of alcohol consumption over a specified period of up to one year. Estimates of alcohol consumption (in standard drinks) are collected monthly by the research team, either in person or by telephone, to reduce recall bias and improve the performance of this method over time [27].

#### Secondary outcomes

Craving is assessed by the Transaddiction Craving Triggers Questionnaire (TCTQ) [28]. This is an instrument designed to assess what triggers craving. The questionnaire is completed at inclusion, M2, M5, and M8. Craving is also assessed using a VAS (0 to 10) at each exposure session [29] and on a monthly basis after M2, in the same way as for the primary outcome.

The frequency of hazardous drinking episodes is assessed with the AUDIT-C (Alcohol Use Disorders Identification Test) questionnaire [30], an abbreviated version of the AUDIT self-report questionnaire [31] that includes three questions about the patient's alcohol consumption. These data are collected at enrollment and at treatment phases M2, M5, and M8. Finally, reported alcohol consumption is correlated with weekly DH toxicology monitoring, if this is routinely performed at the recruiting centers. Weekly toxicology screenings for ethyl glucuronide (EtG), if performed as part of the routine care at the recruiting centers, will be recorded in the logbook.

Anxiety is assessed by the Generalized Anxiety Disorder-7 (GAD-7) questionnaire [32]. This instrument is designed to screen for generalized anxiety disorder and may also help to identify panic disorder, social anxiety disorder, and posttraumatic stress disorder. It is administered at inclusion, M2, M5, and M8.

Depression is assessed by the Patient Health Questionnaire-9 (PHQ-9) [33]. This instrument collects information on the presence and intensity of depressive symptoms. Data is collected at inclusion, M2, M5, and M8.

Self-efficacy is assessed by the Sense of Self-Efficacy Questionnaire, originally developed in German by Matthias Jerusalem and Ralf Schwarzer [34]. It is administered at inclusion, M2, M5, and M8.

Rumination is assessed by the 15-item Perseverative Thinking Questionnaire (PTQ). This is a widely used self-report measure of repetitive negative thinking [35]. It is administered at inclusion, M2, M5, and M8.

The state of presence in virtual reality is measured by the QEP questionnaire (Questionnaire sur l'État de Présence) developed by the UQO Cyberpsychology Laboratory (2002) [36, 37]. This survey is conducted at M2 for the participants randomly assigned to the VR-CET group.

Cybersickness risk is assessed after each VR-CET session using the Cybermalaise Questionnaire from the UQO Cyberpsychology Laboratory [38].

All questionnaires are completed by DH patients at inclusion and M2. The scheduled assessments from M3 to M8 are conducted by telephone (Cf. Table 1).

**Table 1.**
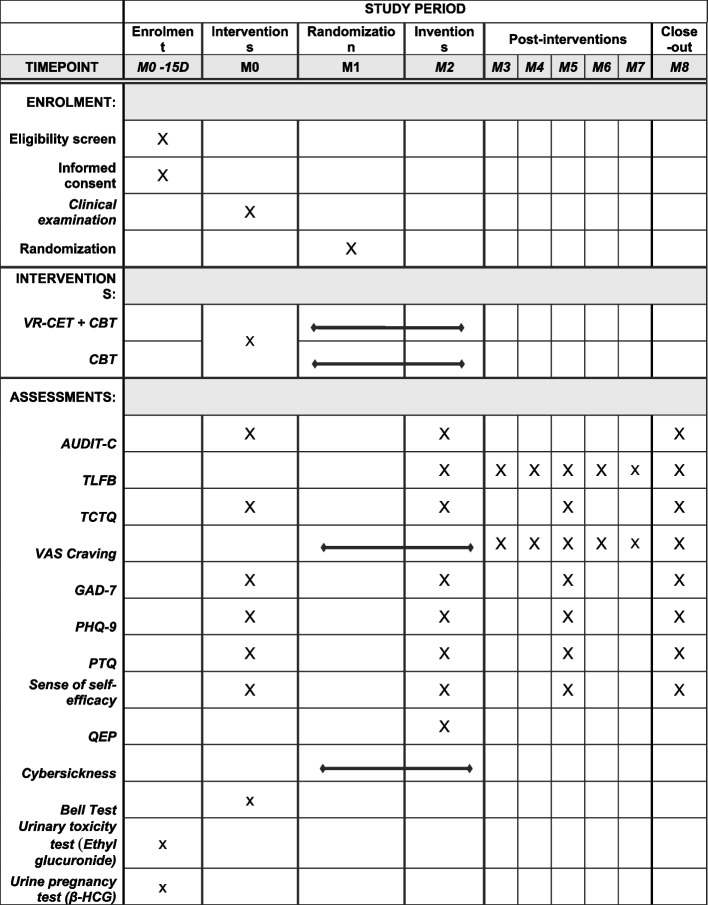
Schedule of enrolment, interventions, and assessments

### Sample size

To determine the number of participants needed for the study, we randomly selected 15 patients with alcohol-related disorders (AUD) who were already being treated at the Albert Chenevier Day Hospital's addiction department. Their cumulative alcohol consumption was monitored at two, five and eight months after admission to estimate the average daily consumption over eight months. The target consumption was calculated on the basis of the recommendations of the European Medicines Agency [39], aiming at a reduction of at least two risk levels according to the World Health Organization. To achieve a statistical power of 0.8, considering a risk α of 0.05 with a two-sided hypothesis, 65 patients per group are required. Assuming a drop-out rate of 20%, 78 patients per group need to be recruited, for a total of 156 patients for both groups. See Table 2 for recruitment procedure.

**Table 2:** Recruitment procedures

	**Number of subjects**
Total number of subjects selected	156
Number of centers	4
Inclusion period (months)	24

### Data collection, management, and analysis

#### Data management

Data are collected on paper and in an observation notebook (with all questionnaires), then entered by the research team on the secure CleanWEB® platform. This platform enables data to be downloaded, anonymized, stored and randomized.

### Statistical methods

The characteristics of all patients will be described globally and according to their randomization group, using the number of patients (%) for qualitative variables and the median [interquartile range] or mean (± standard deviation) for quantitative variables, depending on the normality of their distribution. A patient flow diagram will be generated. Results are reported according to CONSORT recommendations. Tests will be two-tailed, and a significance level of *p* < 0.05 will be considered statistically significant. The primary outcome (self-reported cumulative number of standard drinks consumed at 8 months) will be compared between the two groups using Student's t-test for independent samples. Complementary analyses will be conducted using generalized linear regression models adjusted for minimization factors and number of sessions per setting. Given the repeated measurement of outcomes and the multicenter design of the study, additional analysis will be performed including Cox and mixed models. To consider Survival analysis will also be conducted using a linear mixed model. Secondary endpoints will be analyzed using the same methodology as for the primary endpoint, i.e. a comparison of the mean using the usual tests. primary endpoint, i.e. a comparison of means using standard tests at 8 months. Secondary analyses to assess changes in secondary endpoints will be performed using evaluated using linear mixed models.

### Methods for accounting for missing, unused, or invalid data

Patients who withdraw consent or discontinue follow-up during the study are not replaced. Missing data will be systematically searched for and checked in the patients' medical records. Analyses may be performed after imputation of missing data. Missing data will be handled using multiple imputation as recommended by the National Research Council Panel on Handling Missing Data in Clinical Trials [40].

### Selection of participants for analyses

The intention-to-treat population will include all patients who signed the informed consent form and were randomized to one of the two study arms and will be analyzed according to the initial randomization group. The per-protocol (PP) population will include patients enrolled in the study without major deviations from the current protocol, including erroneous inclusion of patients, changes or noncompliance with the treatment assigned at randomization. The primary endpoint will be analyzed by ITT and secondarily by PP analyses. PP analyses. Secondary endpoints will be analyzed according to ITT and PP principles to ensure robustness of results.

### Data monitoring

Two committees work together to monitor the data and guide the research.

- i) The Steering Committee meets quarterly. Its purpose is to define the general organization, determine the methodology, monitor the progress of the research, coordinate information, initially determine the methodology, validate the applications of persons recruited under the protocol, and define any relevant modifications to the protocol necessary for the continuation of the trial. At the end of the meeting, the sponsor will be informed of any decision that requires rapid action by the sponsor.
- ii) The Scientific Committee meets quarterly. Its purpose is to define the objectives of the protocol, to draft the protocol and to propose modifications during the research. Decisions taken at these meetings are validated after discussion and majority vote.

### Audit

All data, documents and reports may be audited without invoking medical confidentiality. Audits can be carried out at any stage of the research process, by independent persons appointed by the sponsor. The aim is to guarantee the quality of the research, the validity of the results, and compliance with applicable laws and regulations.

### Ethics and dissemination

#### Research ethics approval

This research is conducted in accordance with the World Medical Association's Declaration of Helsinki. The study was approved by the French ethic committee (Comité de Protection des Personnes Sud-Est VI) on 07/07/2023 (National ID 2022-A02797-36). Any substantial modification of the protocol by the coordinating investigator must be approved by the sponsor. Once approved, the sponsor must obtain a favorable opinion from the ethic committee before implementation.

#### Consent or assent

Patients in addictology DH receive initial information from a trained investigator. Informed consent is obtained by the principal investigator, an attending physician, or a qualified person, with a two- to four-week cooling-off period. The investigator documents participation in the patient's medical record, retains a copy of the consent form, and a tamper-proof envelope is archived by the sponsor at the end of the study. No exclusion period is defined at the end of participation. No anticipated harm or compensation to subjects is foreseen, as the research will be organized as part of their routine care. However, participants in the CBT group will be offered the opportunity to receive virtual reality exposure sessions as part of their routine care once participation in the study has ended.

#### Confidentiality and access to data

Quality control personnel ensure confidentiality, making data non-identifiable, using initials and a coded ID. The sponsor ensures that each participant has given written consent to access data. APHP Cleanweb® data will be stored on dedicated servers. Physical documents will be kept in a locked cabinet during the research, then archived in accordance with regulations. Documents specific to minimal-risk research will be archived by the investigator and sponsor for 15 years after the end of the research, including sealed envelopes, files, successive versions of the protocol, ethic committee opinions, correspondence, inclusion list, special appendices, final report, and documents relating to data collection. Study results will be communicated to investigators at all centers, to patients and to the scientific community in general. APHP is the owner of the data. Additionally, a contract has been established with the University of Nanterre. This agreement allows for further statistical analyses to be conducted in collaboration with the CLIPSYD laboratory from the Faculty of Psychology at the University of Nanterre, under the direction of Prof. Lucia Romo. For this purpose, pseudonymized research data will be securely transmitted to the team to facilitate these analyses. All data necessary to support the protocol can be provided upon request to APHP.

## Discussion

The present study aims to evaluate the efficacy of VR-CET combined with CBT in the treatment of AUD in recently abstinent individuals. A recent review of the literature highlights the emergence of virtual reality as an innovative and promising tool in the therapeutic landscape [21]. Studies on the efficacy of VR-CET have mainly focused on craving, with findings such as that exposure to alcohol-related stimuli in virtual reality can increase participants' desire to drink [5]. In terms of its reduction, VR-CET appears to have positive effects, particularly for individuals with AUD [1, 41]. The perceived level of realism of the virtual environment seems to influence this efficacy, with a more pronounced reduction in the desire to drink when the environment is perceived as realistic. About anxiety, the effects of VR-CET were mixed, but integration with CBT resulted in a significant reduction in post-treatment anxiety, although less favorable than in the CBT group alone. About alcohol use, only one study examined this variable and showed that VR-CET had no significant short-term effects [42]. The systematic review highlighted the lack of longitudinal data to consolidate the efficacy of VR-CET as a therapeutic tool to reduce alcohol consumption Table 2.

Our protocol aims to address the limitations of previous research by increasing the sample size (*N *= 156), by aiming not only for abstinence but also for a reduction in consumption, which seems more realistic, by having broad inclusion criteria to allow the inclusion of a population as close as possible to clinical reality, integrating sociodemographic questions into our inclusion questionnaire, and considering comorbidities via the MINI questionnaire at inclusion. In addition, we included urinary toxicity test (ethyl glucuronide) during the two months of therapy to objectify alcohol use data. In response to previous criticism regarding the lack of virtual environments with negative connotations, two neutral environments (subway and supermarket) were included to induce a sense of stress.

Despite these improvements, several limitations must be considered. We did not include the multisensory aspect, in particular olfactory stimulation, which could potentially influence perception of the realism of virtual environments. Furthermore, we did not include objective data such as heart rate or urinary toxins about craving and alcohol consumption, only declarative data are collected, leaving a gap in our understanding of physiological responses to alcohol-related stimuli and on subjects' actual consumption. 

Despite the limitations of our study, *E-Reva* contributes to the literature on the use of VR in the treatment of AUD. These findings could potentially open significant horizons for future patient interventions. VR is emerging as an innovative therapeutic resource that provides a unique setting for exposure to alcohol-related environments. Integrating VR into the treatment of AUD requires a thoughtful approach that considers the specific nuances of this disorder. Personalizing interventions based on a thorough understanding of individual patient needs remains critical to maximizing the therapeutic efficacy of VR. Overall, the steady progress of research in this field promises significant advances. These advances will not only strengthen our understanding of the efficacy of VR in the treatment of AUD but will also open new perspectives for innovative therapeutic interventions focused on the specific needs of patients. Thus, VR is emerging as a promising tool to enrich the therapeutic landscape and pave the way for a more targeted and effective approach to the treatment of AUD.

### Trials status

The study began enrolling participants in October 2023, and recruitment is scheduled to end in October 2025. Protocol version 1.0, 12/05/2023.

### Supplementary Information

Supplementary Material 1.

Supplementary Material 2.

## Data Availability

The data sets analyzed in the current study and the statistical code are available from APHP and the principal investigator on reasonable request, as is the full protocol.

## Abbreviations

AUD: Alcohol use disorder
AUDIT: Alcohol Use Disorders Identification Test
CBT: Cognitive behavioral therapy
CET: Cue exposure therapy
DH: Day hospitals
DSM: Diagnostic and Statistical Manual of Mental Disorders
GAD-7: Generalized Anxiety Disorder 7
PP: Per-protocol
PTQ: Perseverative Thinking Questionnaire
QEP: State of Presence Questionnaire
TCTQ: Transaddiction Carving Triggers
TLFB: Timeline Followback
VAS: Visual analog scale
VR: Virtual reality
VR-CET: Virtual reality cue exposure therapy
