# Supplementary material for: Study on the efficiency of virtual reality in the treatment of alcohol use disorder: study protocol for a randomized controlled trial: E-Reva
Source: Trials. 2024 Jun 27;25:417. doi: 10.1186/s13063-024-08271-x (PMC11212355; doi:10.1186/s13063-024-08271-x)
Supplement: Supplementary file 2 — Supplementary Material 2. [file 13063_2024_8271_MOESM2_ESM.docx]

**APPENDICES**

**
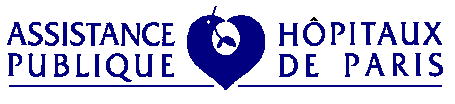
**

**CONSENT FORM**

I, the undersigned, M^me^ , Mr *[delete as appropriate]* (*surname, first name*) .................................................................. **freely agree to take part in the research project entitled "Efficacy of Virtual Reality in the treatment of alcohol use disorder: a multicenter randomized trial" (e-RéVA study)** organized by the Assistance Publique - Hôpitaux de Paris and proposed to me by Doctor (*surname, first name, telephone*) .............................................................................................., the physician involved in this research project.

- I have read the 4-page information note ........... explaining the purpose of this research, how it will be carried out and what my participation will entail,
- I will keep a copy of the information note and the consent form,
- I received appropriate answers to all my questions,
- I've had enough time to make my decision,
- I understand that my participation is voluntary and that I may discontinue it at any time without incurring any liability or prejudice to the quality of the care I receive.
- I have been informed that the data collected in the context of the research may be reused for further research, and that I may object to this at any time.
- I am aware that my participation may also be interrupted by the doctor if necessary, who will explain the reasons for doing so,
- I understand that in order to take part in this research project, I must be affiliated with a social security scheme or be a beneficiary of such a scheme. I confirm that this is the case,
- I have been informed that my participation in this research will last eight months, and that this implies that I will not be able to consider participating in any other research during the entire period of my participation without informing the physician who is treating me for the research,
- my consent in no way relieves the doctor treating me for the purposes of the research or the AP-HP of all their responsibilities, and I retain all my rights as guaranteed by law.

| **Signature of research participant** | **Doctor's signature** |
| --- | --- |
| First name :  Date: Signature : | First name :  Date: Signature : |
